# Sycosis Barbae Treated With Bimekizumab

**DOI:** 10.1111/ajd.14608

**Published:** 2025-10-10

**Authors:** Matiar Madanchi, Riccardo Curatolo, Lorenzo S. Pelloni, Hazem A. Juratli

**Affiliations:** ^1^ Department of Dermatology University Hospital Basel Basel Switzerland; ^2^ Department of Dermatology Ente Ospedaliero Cantonale Bellinzona Switzerland; ^3^ Institute of Medical Genetics and Pathology University Hospital Basel Basel Switzerland

## Abstract

Sycosis barbae (SB) is a chronic, potentially scarring alopecia that primarily affects the beard. We report a unique case of refractory SB in a 31‐year‐old male successfully treated with bimekizumab, a dual inhibitor of interleukin (IL)‐17A and IL‐17F. Significant clinical improvement was observed after the second injection, with complete remission achieved after 4 months, highlighting the potential of this novel therapeutic approach for SB.

**Abbreviations**
BKZ: Bimekizumab
IL: Interleukin
SB: Sycosis barbae

## 1 Introduction

SB is a chronic infection of the hair follicles in the beard and moustache area, typically caused by bacterial or fungal pathogens [1, 2]. Clinically, SB presents as irregular, atrophic white patches of scarring alopecia, follicular pustules or abscess formation. Dermatoscopy reveals tufted hairs, black dots, perifollicular erythema and follicular pustules [1]. The diagnosis is confirmed by clinical findings, bacteriology/mycology cultures and histology. Treatment is challenging and includes topical antiseptics, topical antibiotics and systemic antibiotics [1, 2].

## 2 Case Presentation

**FIGURE 1:**
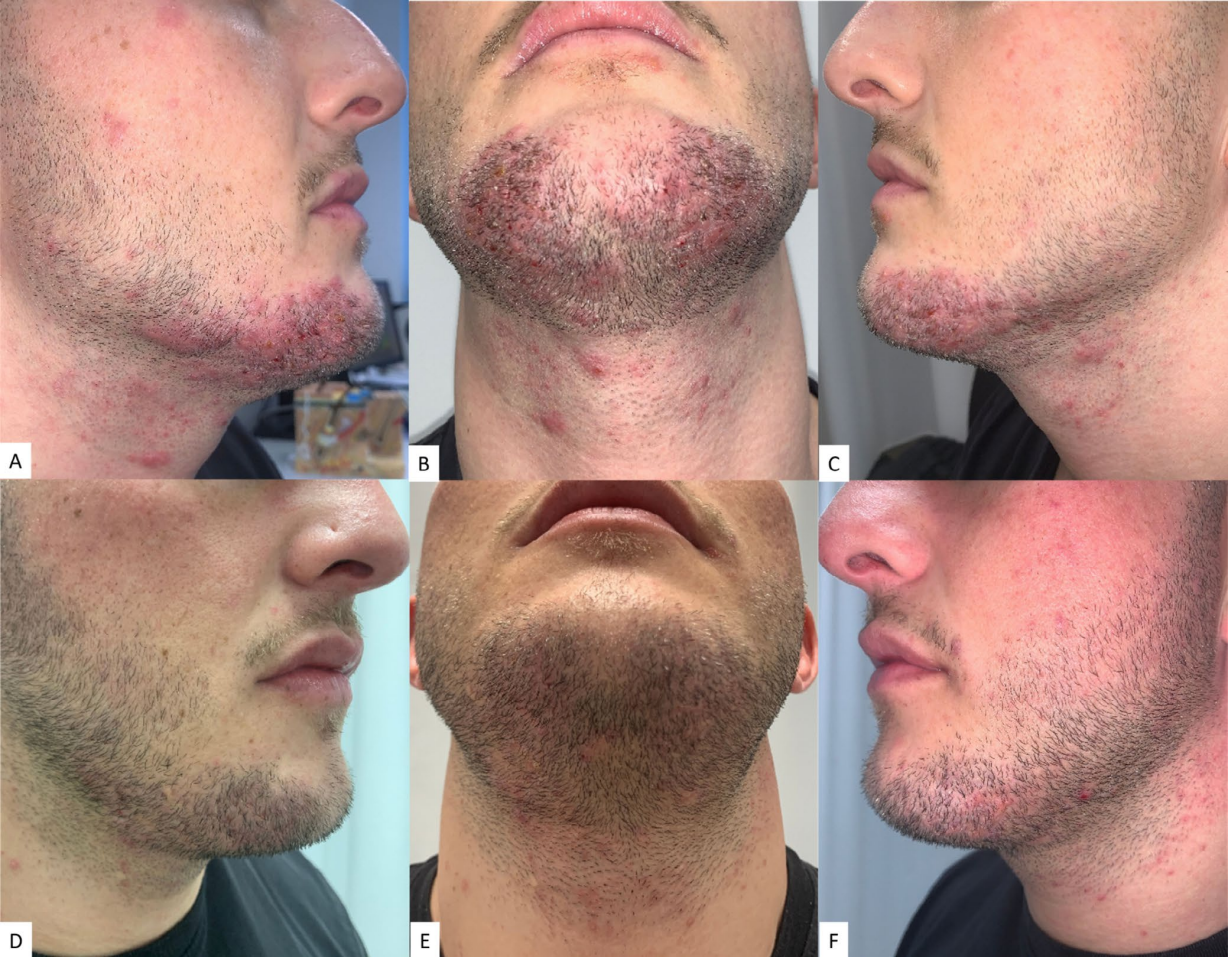
(A–C) Clinical presentation of erythematous plaques and pustules on the entire chin and neck before starting treatment with bimekizumab. (D–F) Clinical presentation after the second injection of bimekizumab showing a significant clinical improvement, reduction in inflammation and pustular presence.

**FIGURE 2:**
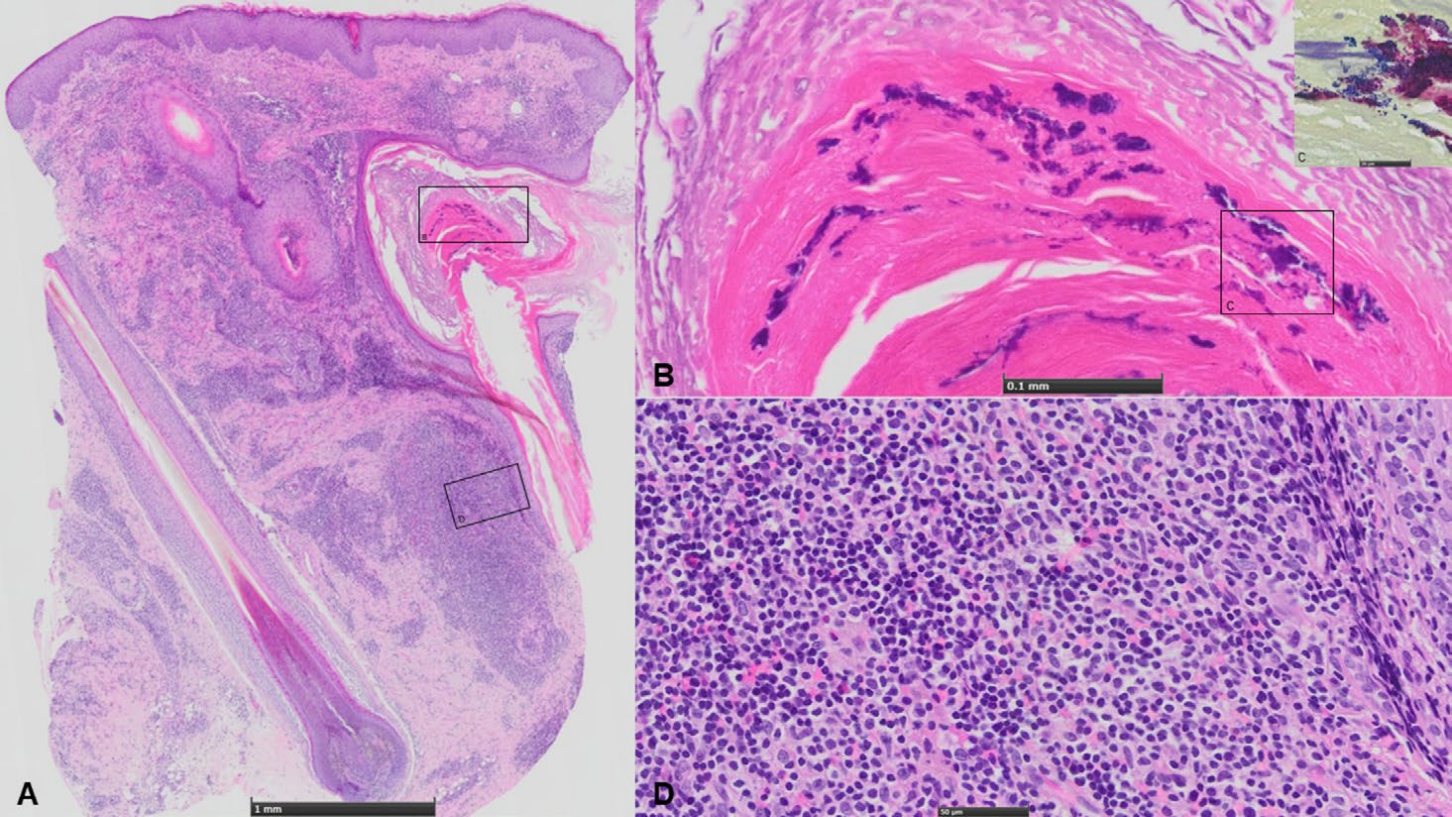
(A) Skin biopsy shows superficial dermal fibrosis with a deep, dense, and diffusely distributed perifollicular infiltrate. The sebaceous glands are destroyed by the inflammatory infiltrate. (B and C) In the dilated infundibula, an intrafollicular accumulation of small cocci, which appear blue on HE staining, highlights numerous Gram‐positive bacterial colonies on Gram staining. (D) The lower part of the hair follicle shows a lymphohistiocytic inflammatory cell infiltrate with plasma cells and eosinophils.

We present the case of a 31‐year‐old healthy male with a 1.5‐year history of erythematous plaques and tufted hairs on the left chin, initially suspected to be bacterial folliculitis and treated with sistemic clarithromycin, isotretinoin and topical fusidic acid without improvement. Subsequent suspicion of tinea barbae led to an ineffective treatment with terbinafine for 1 month. Upon referral to our clinic, he presented with a slightly desquamating erythematous plaque and pustules involving the entire chin and neck (Figure 1A–C). Dermatoscopy confirmed tufted hairs, perifollicular erythema with scale, and scattered follicular pustules. Negative fungal cultures and detection of *Staphylococcus aureus* from a pustule smear led to a skin biopsy. The biopsy revealed a centrally located ruptured follicle surrounded by a deep, dense, and diffusely distributed perifollicular infiltrate composed of numerous lymphocytes, histiocytes, plasma cells, and eosinophils. Gram staining confirmed the presence of Gram‐positive cocci, consistent with the detected *S. aureus* . In addition, superficial dermal fibrosis was observed (Figure 2A–D), leading to the diagnosis of advanced SB linked to recurrent *S. aureus* infection. PAS staining excluded fungal infection. Initial treatment with doxycycline for 3 months and subsequent isotretinoin and dapsone associated with potent topical steroids (betamethasone) showed no improvement and exacerbated clinical symptoms. Therefore, therapy was shifted to bimekizumab (BKZ) (anti‐IL‐17A and anti‐IL17F) 160 mg subcutaneous initially, followed by monthly doses for 4 months and subsequently every 2 months. Significant clinical improvement, reduction of inflammation and pustular presence were noted after the second injection (Figure 1D–F).

## 3 Discussion

SB is a condition that is often triggered by infections caused by various pathogens, including bacteria and fungi. In some ways, however, the pathophysiology of SB may resemble that of folliculitis barbae, whose pathophysiology is not yet fully understood [1]. Various theories have been proposed regarding its pathogenesis, such as the idea that *S. aureus* may act as a superantigen, leading to T cell activation [1, 2]. Research has shown that both IL‐17A and IL‐17F play a role in the host response to extracellular bacteria [3]. Additionally, it is reported that this specific interleukin activates neutrophils, which are often involved in the inflammatory process associated with folliculitis decalvans and SB [3, 4].

To date, there have been reports in the literature of patients with SB linked to recurrent *S. aureus* infection who have been successfully treated with various therapies. However, a treatment strategy that simultaneously inhibits both IL‐17A and IL‐17F has not been tested until now. Based on this hypothesis, we decided to initiate an off‐label treatment with BKZ.

BKZ is a monoclonal IgG1 antibody that selectively inhibits interleukin (IL)‐17F and IL‐17A and is approved for the treatment of plaque psoriasis. In our case, we used the dosage recommended for treating psoriasis. Remarkably, we observed significant improvement of the SB after the second injection, and after approximately 4 months of treatment, the patient achieved complete remission, although some scarring remained.

This may be the first reported case in the literature of refractory SB successfully treated with dual inhibition of IL‐17A and IL‐17F. Further studies are needed to confirm the efficacy of this treatment in SB linked to recurrent *S. aureus* infection patients. Nevertheless, this case may provide insights to better understand the still unclear pathophysiology of this skin condition.

## Conflicts of Interest

The authors declare no conflicts of interest.

## Data Availability

The data that support the findings of this study are available from the corresponding author upon reasonable request.
